# Regular group exercise contributes to balanced health in older adults in Japan: a qualitative study

**DOI:** 10.1186/s12877-017-0584-3

**Published:** 2017-08-22

**Authors:** Hiroko Komatsu, Kaori Yagasaki, Yoshinobu Saito, Yuko Oguma

**Affiliations:** 1 Faculty of Nursing and Medical Care, Keio University, 35 Shinanomachi, Shinjuku-ku, Tokyo, 160-8582 Japan; 2 Graduate School of Health Management and Sports Medicine Research Center, Keio University, 4-1-1 Hiyoshi, Kohoku-ku, Yokohama, Kanagawa 223-8521 Japan

**Keywords:** Older adults, Physical activity, Exercise, Qualitative research, Community

## Abstract

**Background:**

While community-wide interventions to promote physical activity have been encouraged in older adults, evidence of their effectiveness remains limited. We conducted a qualitative study among older adults participating in regular group exercise to understand their perceptions of the physical, mental, and social changes they underwent as a result of the physical activity.

**Methods:**

We conducted a qualitative study with purposeful sampling to explore the experiences of older adults who participated in regular group exercise as part of a community-wide physical activity intervention. Four focus group interviews were conducted between April and June of 2016 at community halls in Fujisawa City. The participants in the focus group interviews were 26 older adults with a mean age of 74.69 years (range: 66–86). The interviews were analysed using the constant comparative method in the grounded theory approach. We used qualitative research software NVivo10® to track the coding and manage the data.

**Results:**

The finding ‘regular group exercise contributes to balanced health in older adults’ emerged as an overarching theme with seven categories (regular group exercise, functional health, active mind, enjoyment, social connectedness, mutual support, and expanding communities). Although the participants perceived that they were aging physically and cognitively, the regular group exercise helped them to improve or maintain their functional health and enjoy their lives. They felt socially connected and experienced a sense of security in the community through caring for others and supporting each other. As the older adults began to seek value beyond individuals, they gradually expanded their communities beyond geographical and generational boundaries.

**Conclusions:**

The participants achieved balanced health in the physical, mental, and social domains through regular group exercise as part of a community-wide physical activity intervention and contributed to expanding communities through social connectedness and mutual support. Health promotion through physical activity is being increasingly emphasized. The study results can help to develop effective physical activity programs for older adults in the community.

**Electronic supplementary material:**

The online version of this article (doi:10.1186/s12877-017-0584-3) contains supplementary material, which is available to authorized users.

## Background

The world is ageing [1]. Japan is the most rapidly aging country in the world, and by 2035, one in every three adults will be aged 65 or above [2]. Caring for a large number of older adults places a great burden on society. Multimorbidity and physical and cognitive disabilities increase with age [3, 4]. Moreover, mental and social health problems are likely to occur with physical health problems [5].

Physical inactivity is recognized as an important risk factor of chronic degenerative diseases and disabilities [6]. Regular physical activity has consistently been shown to improve functional health and has proven effective in preventing or delaying physical and cognitive disabilities [6–8]. Health promotion in older adults is an important public health priority. Maintaining not only physical but also cognitive health is highlighted under the concept of ‘active ageing’, which is the process of optimizing opportunities for health, participation in society, and security for all people as they age, based on the United Nations Principles of independence, participation, dignity, care, and self-fulfilment [1].

Community-wide physical activity interventions have been developed to encourage older adults to stay healthy and active for as long as possible [9, 10]. Recently, community-wide campaigns have been increasing to reduce the health risk factors of entire communities [11]. Community-wide campaigns are characterized by largescale and multicomponent strategies with a key brand message used consistently in all means and channels of communication; these interventions usually involve many sectors and partnerships with high visibility [12]. Although evidence of the effectiveness of community-wide physical activity interventions is limited, some positive results have been reported in the elderly population, including enhanced awareness/knowledge [13], mobility, and strength and improved quality of life [14]. The ‘Fujisawa +10 exercise program’ was developed for older adults as part of a community-wide intervention in Japan [9]. Older adults were encouraged to voluntarily exercise together as a group in their community. Improvement of both physical and cognitive functions has been expected through provision of opportunities to increase physical activity and socializing with peers.

Since the population of older adults is heterogeneous —older adults have varying degrees of health [5], culture, attitudes, and practices [15]—viewing older adults as a homogeneous group and overlooking their differences may hinder individualized health care [16]. Therefore, it is essential to consider older adults’ perspectives to promote their health.

The present study explored the experiences of older adults participating in regular group exercise, which was part of a community-wide intervention, and investigated their perception of the physical, mental, and social changes they underwent as a result of regular group exercise.

## Methods

### Study design

A qualitative study design was used to explore the experiences of older adults who participated in regular group exercise. Focus groups are suited to eliciting participants’ beliefs, attitudes, and feelings through group dynamics encouraged by interactions among participants [17]. These group dynamics constitute the rationale for using focus groups. Interacting with others in group discussion encourages research participants to generate their own views and express their honest feelings, leading to depth and exploration of topics.

Before the focus group interviews were conducted, the study was approved by the Institutional Review Board of the Graduate School of Health Management, Keio University (No. 2015–16). Further, we obtained written and oral informed consent from all participants.

### Community-wide intervention for increasing physical activity (Fujisawa +10)

The community-wide intervention is a multi-level intervention. The intervention originally aimed at increasing physical activity at the population level by encouraging the spread of physical activity among citizens based on the national physical activity recommendations made by the National Institute of Health and Nutrition, which belongs to the Ministry of Health, Labour, and Welfare [18]. The key message of the campaign is ‘Plus Ten (+ 10: Be active for 10 more minutes than now)’. The intervention comprised information, education, and community support provision to raise awareness of the importance of physical activity using media, websites, newsletters, leaflets, and T-shirts with the logo, targeting older adults in partnership with the local government of Fujisawa City and current senior clubs. Since 2014, the community-wide intervention has expanded its activities to prevent dementia. The dementia-related activities include provision of information on the effect of physical activity on dementia prevention, public lectures on physical activity and dementia prevention, regular exercise groups for community-dwelling older adults, and physical and cognitive function measurements among older adults.

### Fujisawa +10 exercise program and regular exercise groups of community-dwelling older adults

We developed the ‘Fujisawa +10 exercise program’ as part of a community-wide intervention [9]. The exercise intensity was low so that the exercises would be suitable for incorporation into an intervention that introduces exercise habits in older adults. The exercise routine consisted of dynamic and static stretching exercises, knee-ups, squatting or knee extensions, arm circle exercises, and body balance exercises to be done in standing or sitting positions. The exercise was introduced to older adults voluntarily exercising together as a group at least once at a city centre or a park in their community. The older adults were taught how to exercise and were given a CD, DVD, and manual as one of tools to do exercise together without an instructor. In addition, they received information on the effect of physical activity on dementia prevention. This group exercise was performed three times a week at a park or community hall in Community A and once a week at a community hall in Community B.

### Participants

The purposively selected participants were older adults participating in the ‘regular group exercise’ in two urban communities (Communities A and B). Two of the investigators, YO and YS, approached the leaders of these two communities. The leaders distributed leaflets containing an overview of the study and the focus group interview to potential participants. Those who were interested in participating voluntarily came to a community hall on the day of the focus group interview, and two investigators (HK and KY) gave the participants an oral and written explanation of the purpose and procedures of the research.

The inclusion criteria for selecting the participants were as follows: the participants (1) were community-dwelling older adults aged 60 or above who were participating in the regular group exercise in Community A or B and (2) lived independently without any elderly care assistance.

## Data collection

### Semi-structured interview guide

Based on literature reviews and discussion among research members on aging and physical activity in older adults, we developed a semi-structured interview guide (Additional file 1).

### Focus group

We conducted four focus group interviews using the semi-structured interview guide between April and June of 2016 at community halls in Fujisawa City. The focus group participants were 26 older adults (11 male and 15 female), and their mean age was 74.69 years (range: 66–86). In Community A, 15 older adults (7 male and 8 female) were grouped into two focus groups with 7 (3 male and 4 female) and 8 (4 male and 4 female) participants. In Community B, 11 older adults (4 male, 7 female) were grouped into two focus groups with 5 (2 male and 3 female) and 6 (2 male and 4 female) participants.

Of these participants, five were living alone. One participant used a stick because of joint pain during the focus group interview, but no participants received formal care. No participants had disabilities or were diagnosed with dementia. Each focus group interview lasted for about 60–80 min. Investigators experienced in conducting focus group interviews (HK and KY) served as facilitators. Two investigators (YO and YS) and two graduate students attended the focus group meetings as observers. The interviews were audio-recorded with the permission of the participants.

## Data analysis

For analyses, we used the constant comparative method in the grounded theory approach [19]. We transcribed the recorded interviews verbatim. The investigators (HK and KY) reviewed the transcripts several times, and personally identifiable information was anonymized. After we collected data pertaining to the first and second interviews, we analysed the data. We continued to collect data, while simultaneously analysing data. After the third and fourth focus group interviews, no new themes emerged, and we confirmed that the data had reached saturation. We analysed the transcripts in the following manner. First, we focused primarily on narratives about physical and cognitive functions and interpersonal relationships in open coding; then, we conducted line-by-line coding. Each meaning was extracted to identify properties and dimensions. Next, we labelled the meanings. For axial coding, we derived subcategories and related them to lead categories. Finally, we identified a core category by relating categories and subcategories. We used qualitative research software NVivo10® to track the coding and manage the data.

To ensure the trustworthiness of the study [20], we adopted the following procedures to establish credibility, dependability, and transferability. One of the investigators (KY) began analysing the data obtained from the first and second focus group interviews in Community A. Then, another investigator (HK) reviewed the data with the codes and themes. Consequently, we conducted the third and fourth focus group interviews, and KY analysed those data. HK reviewed all the codes and themes KY found from the analysis. All the categories and codes were discussed, and final themes were established by KY and HK.

After the analysis, we conducted a peer debriefing to interpret the results with professionals from the fields of geriatrics and mental health. A summary of the study results was provided to the participants as feedback prior to finalizing the main themes and categories; however, no changes were suggested by the participants. After completion of identification of themes and quotations to support themes, a professional translator translated them into English.

The qualitative methods and reporting of results adhered to the Consolidated Criteria for Reporting Qualitative Studies (COREQ) guidelines [21] and Standards for Reporting Qualitative Research (SRQR) [22]. A complete COREQ checklist has been uploaded (Additional file 2).

## Results

### Regular group exercise contributes to balanced health in older adults

**Fig. 1:**
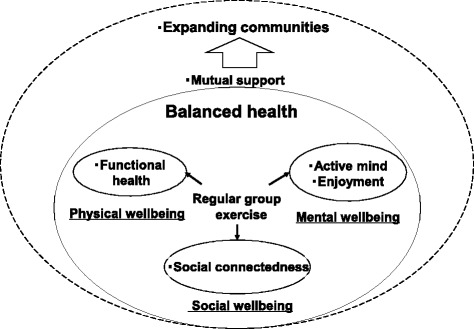
Regular group exercise contributes to balanced health in older adults

In this study, the finding ‘regular group exercise contributes to balanced health in older adults’ emerged as an overarching emergent theme with seven categories (regular group exercise, functional health, active mind, enjoyment, social connectedness, mutual support, and expanding communities) (Fig. 1).

The group exercise contributed to their physical, mental, and social well-being. Although the participants perceived that they were aging physically and cognitively, the regular group exercise helped them to improve or maintain their functional health, socialize with peers, and enjoy their lives. They cared for others and supported each other. Consequently, they felt socially connected and experienced a sense of security in the community. As the older adults began to seek value beyond individuals, they gradually expanded their communities beyond geographical and generational boundaries.

### Regular group exercise

For the older adults, who had plenty of free time after retirement or after they had finished their child-rearing responsibilities, regular group exercise was an important factor in balancing their overall health. Regular group exercise regulated their daily life. Without such a regular plan, they were at risk of irregular habits, such as getting up, sleeping, and eating at irregular hours:

For me, participating in this group exercise helps my daily life. After I retired, I was so free that I often tended to get up late. But when I know it’s an exercise day, I get up on time and get myself ready. I think it’s good for me (73-year-old male, Participant N).

Gathering at one place and exercising with their peers gave them an opportunity not only to participate in physical exercise but also to go out regularly, leading to interaction with others, prevention of isolation, and stimulation in their daily lives. One participant stated, ‘After my children had grown up, I had nothing to do. This group exercise helps regulate my daily life by balancing out the day-to-day tensions and stressors’ (72-year-old female, Participant I). Another participant said, ‘For me, going out is the key. Not just staying home but going out three times a week really helps’ (77-year-old female, Participant C). As for the frequency of regular gathering, a few times a week was well accepted among a majority of the participants. Having off-days motivated them more than having exercise every day.

The participants shared the importance of the group exercise to start each day. One participant emphasized, ‘My day begins with the exercise. My body feels very flexible, and I feel more energized today’ (73-year-old male, Participant N). The regular group exercise provided the older adults with a good routine and helped to build a foundation for daily life. Another participant stated, ‘It helps give my week a sense of structure and routine’ (78-year-old female, Participant Y). The exercise kept them fit, and performing daily activities helped them feel they were in good health.

### Functional health

While the older adults felt elements of aging, including the decline of physical and cognitive functions, many of the participants perceived the regular group exercise as good for their functional health. One participant stated her biological changes:

Now, I feel I am growing older. I used to enjoy intense exercise, but a few years ago, I began experiencing shoulder pain, the so-called ‘shoulder pain for people aged 50’. Of course, I am older than 50 (laughs). Ever since then, I have been unable to do many things (66-year-old female, Participant R).

Other participants talked about the decline of muscle strength and their efforts to improve or at least maintain their current function. One participant stated, ‘People in their 70s are noticeably weaker, particularly their muscles’ (75-year-old female, Participant K). Another participant reported the effect of exercise by stating, ‘Like radio exercise, the +10 exercise has been very effective in improving the muscle strength in my knees’ (73-year-old female, Participant M). Another participant affirmed that it helped maintain her current physical function rather than improve it. ‘It is great if we can do now what we could do at a younger age as we are aging’ (76-year-old female, Participant U).

Decline of appetite and unbalanced nutrition rather than overweight are concerns among Japanese older adults. The participants reported an increase in appetite after the exercise as an indicator of health. One participant stated, ‘I believe that how much you enjoy eating is an indicator of your well-being. Don’t you think so? I really enjoy my meals’ (72-year-old female, Participant I). Other participants agreed with her.

### Active mind

The participants believed that mental health was as important as physical health, and physical function is closely related to cognitive function. On participant stated, ‘I don’t know whether it is related to dementia, but I think that the decline of physical function leads to the decline of cognitive function, and people with poor physical function are likely to become senile’ (73-year-old male, Participant N). Multiple participants agreed with him that the decline of physical health would lead to the decline of cognitive function.

In the +10 exercise, the instructor reminds older adults to try to be aware of which muscle they are using in the exercise. One participant stated, ‘I am not just exercising but am constantly focusing on how each exercise affects each part of the body. Once I know which exercise is for which body part, I try to stretch correctly’ (73-year-old female, Participant M). They felt mentally sharp by focusing on the body action and had a sense of achievement after the exercise.

Furthermore, the participants felt that stimulation as a result of talking to people before and after the exercise would be good for their mental health. They believed that keeping the mind active and talking to peers contributed to maintaining their cognitive function:

I come here and talk to all of you. Like I said before, if I stay at home, I get isolated and have no stimulation. Being with one’s family or watching TV are forms of one-way communication. Talking to people is very stimulating and may help prevent dementia (73-year-old female, Participant M).

### Enjoyment

The participants became more active and began enjoying their lives more. Some of the participants were not willing to go out before joining the regular group exercise. One participant said, ‘When I go out, naturally, I interact with different people. Now, I go out more often. Some people say that they do not want to go out because staying at home is easier. But if you go out, it becomes a habit, and you will enjoy it’ (75-year-old female, Participant K).

The group exercise was an opportunity not just to engage in physical activity but also to socialize. One participant stated, ‘I enjoy coming here’ (73-year-old female, Participant M). Another participant expressed, ‘I joined the exercise, and we got to know one another. Now, I can talk to you as friends. It really makes me happy’ (77-year-old female, Participant C). For the participants, talking to their peers and exchanging information with them stimulated them, and, in the process, they also laughed and shared jokes with their peers. One participant stated, ‘Yes, the laughter…they say that laughter is good for the health. I believe it is good for us, too’ (76-year-old female, Participant U).

Exercising alone at home could never have brought about this type of enjoyment. Moreover, the group exercise motivated the participants to continue engaging in physical activity. One participant stated, ‘I can hardly wait for Monday. If I have something to do, I try to finish it or rearrange my schedule, because I really look forward to Mondays’ (79-year-old female, Participant Z). As one participant said, ‘I really enjoy it. That’s why I am able to continue. It’s fun’ (79-year-old male, Participant Q). Most participants attend the exercise for enjoyment, and, furthermore, they felt connected.

### Social connectedness

By participating in the regular group exercise, the participants felt that they were socially connected and that they were members of a society. One participant stated, ‘When we talk to each other, we feel connected’ (77-year-old female, Participant C).

Eventually, the participants expanded their communication beyond the group exercise. They planned social activities with their peers, including travels and eating out:

We make many plans. We are proactive when it comes to going out, and we take trains and buses. Even though we are older, it stimulates us. When we go out, we care about how we dress, which is also good (73-year-old female, Participant M).

With increased social activities, the older adults began to actively connect with others. One participant stated, ‘I get stimulated in various ways. I feel like trying. For example, I want to be creative in my cooking. If I cook well, I feel like sharing my dish with someone’ (72-year-old female, Participant I). The social connectedness is not limited to sharing enjoyment with peers but led to the momentum to build a community to care and support for each other.

### Mutual support

The regular group exercise helped the older adults to develop their potential and respect each other. One participant emphasized the value of older adults by stating, ‘As we grow old, we all have our strong points’ (75-year-old female, Participant K). Other participants affirmed by saying, ‘Each of us is good at something’ (73-year-old female, Participant M), and ‘We do what we are good at’ (72-year-old female, Participant I).

As for physical and mental well-being, and social participation were increased among older adults; they began to care for their peers and other people in the community. They supported each other and felt a sense of security in the community. One participant stated, ‘(During the exercise,) I look around (laughs). If someone is absent, I wonder what happened to him or her because he or she is always present. So, to check on the absent person, I call him/her on the way home’ (75-year-old female, Participant K).

Because of aging, many older adults face late-life challenges and are concerned about the future. They felt a sense of security when they found that not only their families but also people in the community cared about them:

These interactions that I have with my peers are very helpful, because these are people I can go to for advice and who help me, even though they do not necessarily live close by. They are a source of psychological support for me, and I really appreciate it (73-year-old female, Participant M).

### Expanding communities

While the participants respected and supported each other in the community, they gradually expanded communities. They perceived that they became more generous and open toward others. One participant stated, ‘People are sweet and have big hearts’ (76-year-old female, Participant U), and another said, ‘Everybody seems to be in good spirits’ (76-year-old male, Participant A).

Multiple participants shared their acceptance of others. One participant stated, ‘Everyone is nice enough to accept everyone’ (77-year-old female, Participant C). Another participant stated, ‘We accept everybody (laughs)’ (75-year-old female, Participant K). They realized that their community was expanding. One participant stated, ‘The community has really expanded naturally, from one neighbouring town to the other. I wonder how many new members have joined us’ (76-year-old male, Participant A). The group exercise in this community is not limited to older residents in the community; it is also open for neighbouring communities.

In recent times, older adults in other communities have seen or heard about the participants’ lively activities. As the number of participants increased to include people living beyond the community boundary, the wave of exchange has expanded:

People were hesitant to join us because they assumed it would be a gathering of older people. But we are active and enjoy many things. Once people realized this, even those younger than 60 wanted to join us (73-year-old female, Participant M).

This change was expressed by one participant as follows: ‘We can see the flower this much, but the roots are expanding, aren’t they?’ (78-year-old male, Participant V). The regular group exercise worked as a catalyst to expand communities beyond geographical and generational boundaries, and the participants sought to strengthen connections in a wider circle of people.

## Discussion

The results of this study give us new insights into the effects of community-wide physical activity intervention among older adults. The finding ‘regular group exercise contributes to balanced health in older adults’ emerged as an overarching theme with seven categories (regular group exercise, functional health, active mind, enjoyment, social connectedness, mutual support, and expanding communities). Balanced health implies physical, mental, and social well-being consistent with the definition of health [23]. The benefits of functional health, an active mind, enjoyment, and social connectedness derived from regular group exercise were found to be important elements of the older adults’ perceived health. The older adults felt a sense of security through supporting each other and gradually expanded communities.

A recent systematic review of qualitative studies of older adults’ perspectives on participation in physical activity revealed that they enjoyed the activity, gained a sense of belonging, and forged friendships by seeing familiar faces or neighbours while performing the exercise, and this motivated them to continue [24]. Being surrounded by people and feeling a sense of connection and that somebody cares have been reported by older adults as important benefits of group exercises [25]. Our study added the understanding of physical activity that contributed to the balanced health of individuals and expanding communities through social connectedness and mutual support.

As aging takes place within the context of others, including family members, friends, and neighbours, social connections are as important as physical and mental health [1]. As many of the older adults were motivated to attend the group exercise as it gave them a sense of social connectedness, it is evident that physical activity often serves as an important means of socialization [26]. Halaweh et al. reported that social connectedness contributed to the status of staying active, functioning, and maintaining one’s own role [27]. The results of our study were consistent with their results that this connectedness was based on older adults’ perceptions that they are still members of a society and willing to contribute to the society. Just as emotionally balanced people are likely to forgive others [28], the participants in this study stated that they had become more generous and open toward others and tried to reach out to vulnerable people. Austin et al. stated that community capacity-building enables older adults to play a role in the building of a community, and, in doing so, older adults can succeed in not only promoting their health but also helping others in the caring community [29].

In recent times, ‘the neighbourhood connection’ has become the focus of global attention when creating hospitable and supportive environments for older adults [29]. In Japan, a three-generation family was traditionally common in which older adults lived with their children and grandchildren. Today, however, social isolation of older adults is an emerging challenge with an increase in the number of nuclear families. Japan has a national plan called ‘the Integrated Community Care’ that aims at creating a community in which people, including those with disabilities and dementia, are able to live in familiar settings throughout their lives [30]. Expanding communities produces new relationships and intergenerational support that is the foundation of the Integrated Community Care.

The group exercise gave the retired older adults a good daily routine and helped them develop and stick to healthy habits. Centre-based programs have resulted in good adherence to physical activity in the short term and also have positive and significant effects on behaviour change [31]. Gathering at one place and exercising with their peers provided the elderly adults with opportunities not only for physical activity but also for going out, socializing, and stimulation in daily life.

Not sporadic but regular and not home-based individual but centre-based group exercise contributed to the balanced health of the older adults. ‘Regular’ and ‘group’ were the factors that were strongly associated with attending the regular group exercise. The participants were motivated by their perceived obligation toward the group, which is consistent with the literature [26]. There is a possibility, however, that some of the older adults who were not interested in the group exercise but participated because of fixed scheduling were excluded from this study because the focus group interview was voluntary.

Maintaining functional independence is the key goal for older adults. The rationale behind physical activity recommendations is the minimization of age-related changes in physiology because such changes put the individual at risk for developing diseases and disability [31]. The older adults were aware of their increasing physical limitations. Some of the participants realized that physical activity improved their physical functions while others emphasized that it served to maintain the current level of their physical functions. The perceived value of physical activity is an important factor in the acceptability of physical activity interventions, and this acceptability is increased when an individual experiences personally relevant benefits from the activity [26].

The benefits of improved physical and mental well-being are well known in older adults [31–34]. Interestingly, while exercising, the participants strived to be aware of each exercise action. They believed keeping their minds active helped to maintain their cognitive function and prevent dementia. In 78% of the studies reviewed by Franco et al. [24], an immediate improvement in mental alertness was reported, and participants believed that physical activity could prevent mental illness. Exercise is associated with the attenuation of age-related mental decline and the preservation of mental capacities [35]. Keeping one’s mind active in addition to engaging in physical activity is important to maintain cognitive health [34]. In our study, the participants perceived psychological benefits of physical activity, including increased feeling of energy and improved self-confidence, and actively sought to keep their minds active. These results suggest that physical activity programs making older adults conscious of psychological benefits would be effective for health promotion.

A positive and optimistic attitude toward life is fundamental for successful aging [16, 25, 28]. Physical activity has positive effects on happiness and its dimensions, including self-efficacy, self-esteem, positive mood, and mental health [36]. In this study, physical activity triggered behaviour changes in the older adults; they went out more often, became more active, and enjoyed their lives. Consistent with other studies [16, 25, 26], enjoyment is often cited by participants, and this element of enjoyment is strongly related to social interactions.

The results of the study revealed that the physical activity was well accepted by older adults as a community-wide intervention, and it contributed to their balanced health. The outcomes of the intervention should be longitudinally measured, and barriers should be identified in a follow-up study. Since this intervention has been designed as part of a dementia prevention project, quantitative assessments of the intervention’s effects on dementia are currently underway. This concept of balanced health can be used when training public health professionals and health care providers to promote health and provide physical, mental, and social support to older adults.

## Strengths and limitations

This study has several limitations that need to be considered. Since this study uses the purposeful sampling, there may be selection bias. In this study, we only recruited older adults who participated in the Fujisawa +10 exercise program; we did not recruit non-participants. Therefore, the results might be biased toward motivated participants. In the future, we should include older adults who do not participate in the physical activity. Their views may differ from those of the participants in this study. The themes were founded on the perspectives of a relatively small sample (*n* = 26), and the effect of the difference in the proportion of male versus female participants (11:15) may have influenced the results. Further, we did not collect socioeconomic data; therefore, socioeconomic perspectives are lacking.

Despite these limitations, this study is one among the few qualitative studies on community-wide physical activity interventions for older adults, although, recently, the number of qualitative studies on physical activity in older adults has been increasing. The results of the study could help to promote physical activity and health among older adults and aid in the development of public health policies in multiple domains from the perspectives of older adults.

## Conclusions

Regular group physical activity contributes to physical, mental, and social health and well-being of community-dwelling older adults and contributes to expanding communities beyond geographical and generational boundaries through social connectedness and mutual support. The aging population demands national action, and health promotion through physical activity is increasingly being emphasized. Our study can help in the development of effective physical activity programs for older adults in the community.

## Additional files

Additional file 1: Interview guide. (DOCX 15 kb)

Additional file 2: COREQ guidelines. (DOCX 21 kb)
