# Supplementary material for: Regular group exercise contributes to balanced health in older adults in Japan: a qualitative study
Source: BMC Geriatr. 2017 Aug 22;17:190. doi: 10.1186/s12877-017-0584-3 (PMC5567431; doi:10.1186/s12877-017-0584-3)
Supplement: Supplementary file 1 — Interview guide. (DOCX 15 kb) [file 12877_2017_584_MOESM1_ESM.docx]

**Additional file 1　　Interview guide**

1. Please introduce yourself.
2. What change have you noticed in your body, memory, activity or daily living through participating and continuing the Fujisawa +10 exercise program?
3. Have you experienced any internal changes (e.g., feelings and values) as a result of the regular group exercise?
4. Have you ever felt that the physical activity was effective to prevent cognitive impairment (e.g., memory loss)? Have you noticed any change?
5. How have the perceived changes in yourself affected your interpersonal relationships or social interactions?
6. Have you developed an interest or concern about others through interaction with different people?
7. Do you think that any change is occurring among the group or community?
8. Do you have any comments on future directions for this activity?
