# Supplementary material for: Regular group exercise contributes to balanced health in older adults in Japan: a qualitative study
Source: BMC Geriatr. 2017 Aug 22;17:190. doi: 10.1186/s12877-017-0584-3 (PMC5567431; doi:10.1186/s12877-017-0584-3)
Supplement: Supplementary file 2 — COREQ guidelines. (DOCX 21 kb) [file 12877_2017_584_MOESM2_ESM.docx]

**Additional file 2: Consolidated criteria for reporting qualitative studies (COREQ): 32-item checklist**

**Title：Regular Group Exercise Contributes to Balanced Health in Older Adults in**

**Japan: A Qualitative Study**

**Authors:** Hiroko Komatsu, RN, PhD., Kaori Yagasaki, RN, PhD., Yoshinobu Saito, PhD., Yuko Oguma, MD, PhD

| **No. Item** | **Guide questions/description** | **Response** |
| --- | --- | --- |
| **Domain 1: Research team and reflexivity** | | |
| *Personal Characteristics* | | |
| 1. Interviewer/facilitator | Which author/s conducted  the interview or focus group? | Hiroko Komatsu (HK), and Kaori Yagasaki　(KY). |
| 2. Credentials | What were the researcher’s  credentials? E.g. PhD, MD | HK: PhD, Registered Nurse (RN), Professor, Dean.  KY: PhD, Registered Nurse (RN), Associate Professor.  YS: PhD, Health Fitness Programmer.  YO: PhD, Medical Doctor (MD), Sports Medicine, Associate Professor. |
| 3. Occupation | What was their occupation at the time of the study? | Researcher and faculty. |
| 4. Gender | Was the researcher male or  female? | Three females and one male. |
| 5. Experience and training | What experience or training  did the researcher have? | HK and KY have done previous several qualitative research projects.  YO and YS have current community-based research projects, and have physical activity promote projects and research. |
| *Relationship with participants* | | |
| 6. Relationship established | Was a relationship  established prior to study  commencement? | HK and KY had no relationships with the participants prior to the study, while YO and YS knew the leaders of two communities through another community based research. |
| 7. Participant knowledge of the interviewer | What did the participants  know about the  researcher? e.g. personal  goals, reasons for doing the  research | Participants knew about the two interviewers’ names and facility. |
| 8.Interviewer characteristics | What characteristics were  reported about the  interviewer/facilitator? e.g.  bias, assumptions, reasons  and interests in the  research topic | Participants did not know about interviewers’ characteristics except their names and facility. |

| **Domain 2: study design** | | |
| --- | --- | --- |
| *Theoretical framework* | | |
| 9. Methodological orientation and Theory | What methodological  orientation was stated to  underpin the study? e.g.  grounded theory, discourse  analysis, ethnography,  phenomenology, content  analysis | See manuscript in the method section/study design. |
| *Participant selection* | | |
| 10. Sampling | How were participants  selected? e.g. purposive,  convenience, consecutive,  snowball | See manuscript in the method section/participants. |
| 11. Method of approach | How were participants  approached? e.g. face-to face, telephone, mail, email | See manuscript in the method section/participants. |
| 12. Sample size | How many participants were in the study? | 26 participants.  See manuscript in the method section/data collection and results section. |
| 13. Non-participation | How many people refused  to participate or dropped out? Reasons? | No one has dropped out. |
| *Setting* | | |
| 14. Setting of data collection | Where was the data collected? e.g. home, clinic,  workplace | Two community centers in Fujisawa city, Kanagawa prefecture.  See manuscript in the method section/data collection. |
| 15.Presence of nonparticipants | Was anyone else present  besides the participants  and researchers? | Yes. Two (female): PhD – students. |
| 16. Description of sample | What are the important  characteristics of the sample? e.g. demographic data, date | See manuscript in the method section/participant and focus group, and results. |
| *Data collection* | | |
| 17. Interview guide | Were questions, prompts,  guides provided by the  authors? Was it pilot  tested? | There was no pilot testing.  See manuscript in the method section/  data collection/semi-structure interview guide. |
| 18. Repeat interviews | Were repeat interview  carried out? If yes, how  many? | No. |
| 19. Audio/visual recording | Did the research use audio  or visual recording to  collect the data? | Data was audio recorded.  See manuscript in the method section/  data collection. |
| 20. Field notes | Were field notes made  during and/or after the  interview or focus group? | No. |
| 21. Duration | What was the duration of  the interviews or focus  group? | Approximately 60-80 minutes.  See manuscript in the method section/  data collection. |
| 22. Data saturation | Was data saturation  discussed? | Yes. See manuscript in the method section/data collection. |
| 23. Transcripts returned | Were transcripts returned  to participants for comment  and/or correction? | No. |

| **Domain 3: analysis and findings** | | |
| --- | --- | --- |
| *Data analysis* | | |
| 24. Number of data coders | How many data coders coded the data? | Two, KY and HK. |
| 25. Description of the coding tree | Did authors provide a  description of the coding tree? | No. |
| 26. Derivation of themes | Were themes identified in  advance or derived from the data? | See manuscript in the method section/  data analysis. |
| 27. Software | What software, if applicable, was used to manage the data? | NVivo 10^®^ software was used to coded and manage our data. |
| 28. Participant checking | Did participants provide  feedback on the findings? | Yes.  See manuscript in the method section/  data analysis. |
| *Reporting* | | |
| 29. Quotations presented | Were participant quotations  presented to illustrate the  themes/findings? Was each  quotation identified?  e.g. participant number | Yes. See manuscript in the results. |
| 30. Data and findings  consistent | Was there consistency  between the data presented and the findings? | Yes. See manuscript in the data analysis/the trustworthiness of the study. |
| 31. Clarity of major themes | Were major themes clearly  presented in the findings? | Yes. See manuscript in the results. |
| 32. Clarity of minor themes | Is there a description of diverse cases or discussion of minor themes? | Yes. See manuscript in the results. |

Developed from: Tong A, Sainsbury P, Craig J. Consolidated criteria for reporting qualitative research (COREQ): a 32-item checklist for interviews and focus groups. *International Journal for Quality in Health Care*. 2007. Volume 19, Number 6: pp. 349 – 357
